# Pine Bark and Green Tea Concentrated Extracts: Antioxidant Activity and Comprehensive Characterization of Bioactive Compounds by HPLC–ESI-QTOF-MS

**DOI:** 10.3390/ijms151120382

**Published:** 2014-11-06

**Authors:** María de la Luz Cádiz-Gurrea, Salvador Fernández-Arroyo, Antonio Segura-Carretero

**Affiliations:** 1 Department of Analytical Chemistry, University of Granada, c/Fuentenueva s/n, 18071 Granada, Spain; E-Mail: mluzcadiz@ugr.es; 2 Research and Development of Functional Food Centre (CIDAF), PTS Granada, Avda. Del Conocimiento s/n, Edificio BioRegion, 18016 Granada, Spain; 3 Biomedical Research Centre, University Hospital of Sant Joan, IISPV, Rovira i Virgili University, C/Sant Joan s/n, 43201 Reus (Tarragona), Spain; E-Mail: sfernandez@fiispv.cat

**Keywords:** pine bark, green tea, polyphenols, flavan-3-ols, procyanidins, antioxidant activity, HPLC–ESI-QTOF-MS

## Abstract

The consumption of polyphenols has frequently been associated with low incidence of degenerative diseases. Most of these natural antioxidants come from fruits, vegetables, spices, grains and herbs. For this reason, there has been increasing interest in identifying plant extract compounds. Polymeric tannins and monomeric flavonoids, such as catechin and epicatechin, in pine bark and green tea extracts could be responsible for the higher antioxidant activities of these extracts. The aim of the present study was to characterize the phenolic compounds in pine bark and green tea concentrated extracts using high-performance liquid chromatography coupled to electrospray ionization mass spectrometry (HPLC–ESI-QTOF-MS). A total of 37 and 35 compounds from pine bark and green tea extracts, respectively, were identified as belonging to various structural classes, mainly flavan-3-ol and its derivatives (including procyanidins). The antioxidant capacity of both extracts was evaluated by three complementary antioxidant activity methods: Trolox equivalent antioxidant capacity (TEAC), ferric reducing antioxidant power (FRAP) and oxygen radical absorbance capacity (ORAC). Higher antioxidant activity values by each method were obtained. In addition, total polyphenol and flavan-3-ol contents, which were determined by Folin–Ciocalteu and vanillin assays, respectively, exhibited higher amounts of gallic acid and (+)-catechin equivalents.

## 1. Introduction

Medicinal and spice plants, which are well known for their pharmacological activity, contain many substances that exhibit radical-scavenging properties. Phenolic compounds are among the other substances included in this group. These compounds, which are secondary plant metabolites, are an essential part of the human diet. They are of considerable interest, due to their suggested advantageous health effects and possibility for use as natural food additives, since they influence the quality and stability of foods by acting as flavorants, colorants and antioxidants [1]. Great interest is currently centered on their potential benefits as complements to the organism’s antioxidant defense system. Polyphenols are potent free radical-scavengers and are associated with multiple biological activities, including radioprotective, anti-inflammatory, anti-carcinogenic, antiviral and antibacterial properties, which are mainly attributed to their antioxidant and antiradical activity [2,3,4]. The *in vitro* antioxidant activity of foods and plants is generally studied by Trolox equivalent antioxidant capacity (TEAC), 2,2-diphenylpicrylhydrazyl (DPPH), ferric reducing antioxidant power (FRAP) and oxygen radical absorbance capacity (ORAC)-based methods [5,6,7]. Different methods are used for total phenolic and flavan-3-ol content determination, with the most common being the vanillin assay [8,9] and Folin–Ciocalteu assay [10].

**Figure 1:**
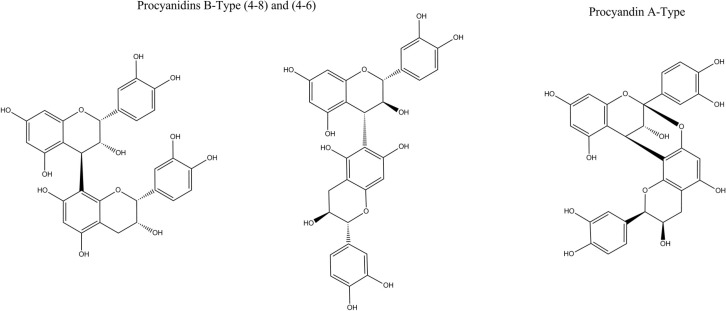
Structures of procyanidin (4β→8) and (4β→6)-dimers (B-type) and the (2β→7, 4β→8)-dimer (A-type).

Proanthocyanidins are found in many woody plants. The two most common sources of them are grape seeds (*Vitis vinifera*) and white pine (*Pinus maritima*, *Pinus pinaster*). Proanthocyanidins are also abundant in green tea (*Camellia sinensis*) and hawthorn (*Crataegus oxyacantha*), as well as in apples, berries, barley, bean hulls, cacao beans, rhubarb, rose hips and sorghum. These compounds are oligomers and polymers of flavan-3-ol monomer units most frequently linked either as C4→C6 or C4→C8 (B-type proanthocyanidins). A-type proanthocyanidins possess a second interflavanoid bond, resulting in oxidative coupling between the C2→O7 positions (Figure 1). The most common classes are procyanidins consisting of catechin, epicatechin and/or their gallic acid esters and prodelphinidins containing gallocatechin and epigallocatechin and/or their galloylated derivatives [11,12].

Pine (*Pinus sylvestris* L.) tree bark is also valued medicinally for its rich content of proanthocyanidins. Pine bark extracts have been used as a folk medicine and are used as a dietary supplement and phytochemical remedy for several diseases (pycnogenol) [13,14]. They have also been shown to be a very powerful antioxidant and free radical-scavenger, even more powerful than either vitamin C or vitamin E. Pine bark extract is used in cardiovascular and heart formulas and has also been shown to be beneficial to those with chronic venous insufficiency. Procyanidins occurring in pine bark consist mainly of the flavan-3-ol units of (+)-catechin [15,16].

Aside from water, tea (*Camellia sinensis*) is the most consumed beverage in the world. About 2.5 million tons of tea are produced worldwide every year. The most popular type of tea in the world is black tea, which is produced in India, Sri Lanka, Kenya and many other countries. Most of the tea produced in Japan is green tea, while China produces both green and black teas, as well as several other types of teas, such as oolong tea and Pu-er tea. In fresh tea leaves and green teas, catechins are the major polyphenols and are mainly composed of (−)-epicatechin, (−)-epigallocatechin, (−)-epicatechin gallate and (−)-epigallocatechin gallate [17,18].

Bioactive compounds have been analyzed by gas-chromatography coupled to mass spectrometry (GC–MS), high-performance liquid chromatography (HPLC) and capillary electrophoresis (CE). HPLC and CE allow for efficient separation of flavonoids in different plant extracts. Electrospray ionization mass spectrometry (ESI-MS) allows for a softer ionization and permits structural information to be obtained using collisionally-induced dissociation (CID). Moreover, ESI-MS makes it possible to discriminate between various flavonoid classes and gather information on the glycosylation position [19]. The negative ion ESI mass spectra show the presence of a series of non-galloylated and galloylated oligomeric procyanidins up to a trigalloylated octamer [20]. Reversed-phase high performance liquid chromatography (RP-HPLC) coupled to diode array detection (DAD) and/or MS are usually employed for analysis of these compounds [6,7,21,22]. Quadrupole time-of-flight mass spectrometry (QTOF-MS) combines high sensitivity and mass accuracy for both precursor and product ions, providing the elemental composition of the parent and fragment ions. This feature helps to identify compounds thoroughly and to differentiate between isobaric compounds. The potential of HPLC–ESI-QTOF-MS for qualitative purposes has been highlighted in several studies [23].

In this work, procyanidin-rich extracts from pine bark and green tea were analyzed and compared by HPLC coupled to a quadrupole time-of-flight (QTOF) mass spectrometer and equipped with an ESI interface. Additionally, we wanted to determinate the antioxidant potential present in both extracts by three complementary antioxidant activity methods: TEAC, FRAP and ORAC. We also wanted to evaluate the total phenolic and flavan-3-ol contents by Folin–Ciocalteu and vanillin assays.

## 2. Results and Discussion

### 2.1. Characterization of Polar Compounds by High-Performance Liquid Chromatography Coupled to Electrospray Ionization Mass Spectrometry (HPLC–ESI-QTOF-MS)

A comprehensive characterization of phenolic compounds using advanced and powerful techniques is crucial. For this reason, suitable methods need to be established for their characterization in vegetable matrices. The use of QTOF technologies allows for the exact mass measurements of both MS and MS/MS ions to be achieved, which is essential for elemental composition assignment and, thus, for the characterization of small molecules [6,7].

#### 2.1.1. Pine Bark Extract

**Figure 2:**
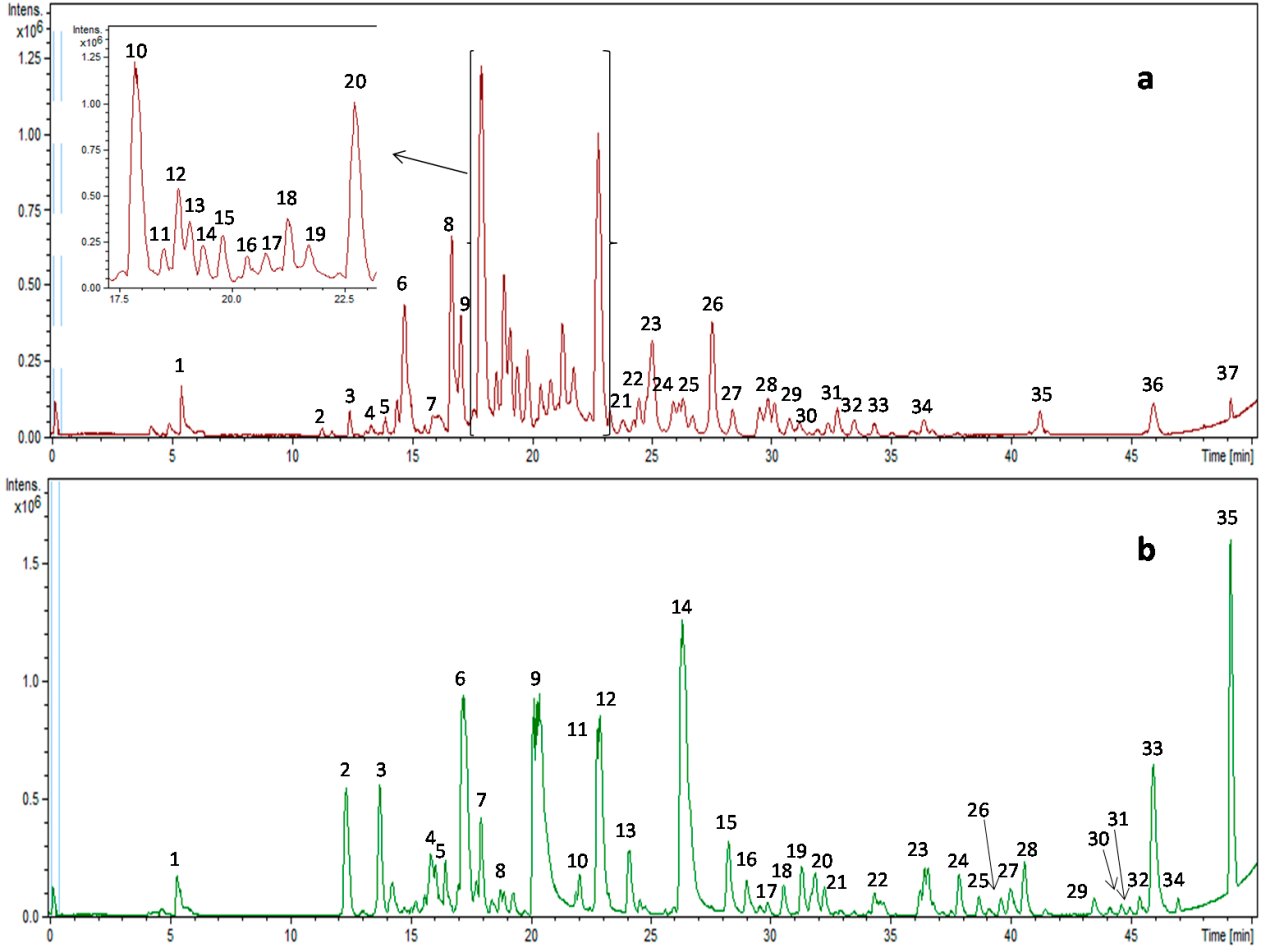
Base peak chromatogram of pine bark (**a**) and green tea (**b**) extracts.

A total of 37 compounds distributed in three major categories (flavan-3-ol and its derivatives, flavonols and other compounds) were analyzed in the present study. Figure 2a shows the base peak chromatogram (BPC) of the pine bark extract. The major peaks, which were identified based on elution order, are listed in Table 1. All of the compounds were characterized by interpretation of their mass spectra obtained by the QTOF-MS and also by taking into account previously reported data.

##### Flavan-3-ol and Its Derivatives

Pine bark, which is valued medicinally for its rich content of proanthocyanidins, has been used as a folk medicine and is used as a dietary supplement. The main constituents of pine bark are known to be phenolic compounds, broadly divided into monomers (catechin, epicatechin) and condensed flavonoid (procyanidins) [13,24,25]. Procyanidins consist mainly of the flavan-3-ol units of (+)-catechin [15].

**Table 1:** Retention time and mass spectral data of the compounds characterized in pine bark extract by HPLC–ESI-QTOF-MS and MS/MS in negative mode.

Peak	Proposed Compound	*RT* (min)	[M–H]^−^ Measured	[M–H]^−^ Calculated	Error (ppm)	mSigma	Fragmentation Pattern	Molecular Formula	Ref.
1	Sucrose	5.4	3,411,098	3,411,089	2.6	9	non fragmented	C_12_H_22_O_11_	–
2	Procyanidin C	11.3	8,651,981	8,651,985	0.5	30	577, 289	C_45_H_38_O_18_	[1,13,15,24,25,26,27]
3	Gardenoside	12.4	4,031,257	4,031,246	2.7	6.2	non fragmented	C_17_H_24_O_11_	–
4	Procyanidin A (isomer 1)	13.3	5,751,191	5,751,195	0.6	22	289	C_30_H_24_O_12_	–
5	Procyanidin A (isomer 2)	13.9	5,751,189	5,751,195	1	34.8	289	C_30_H_24_O_12_	–
6	Procyanidin B (isomer 1)	14.7	5,771,366	5,771,351	2.6	5.3	425	C_30_H_26_O_12_	[1,13,15,24,25,26,27]
7	Procyanidin B (isomer 2)	15.9	5,771,347	5,771,351	0.7	13.2	425, 289	C_30_H_26_O_12_	[1,13,15,24,25,26,27]
8	Chalcan-flavan-3-ol dimer (isomer 1)	16.7	5,791,532	5,791,508	4.2	5.2	561	C_30_H_28_O_12_	–
9	Procyanidin trimer A-type (isomer 1)	17	8,631,842	8,631,829	0.4	27.4	289, 285	C_45_H_36_O_18_	–
10	(−)-epicatechin	17.9	2,890,727	2,890,718	3.4	4.9	245	C_15_H_14_O_6_	[1,13,15,24,25,26,27]
11	Chalcan-flavan-3-ol dimer (isomer 2)	18.5	5,791,512	5,791,508	0.7	4.3	289	C_30_H_28_O_12_	–
12	Chalcan-flavan-3-ol dimer (isomer 3)	18.8	579,152	5,791,508	2.1	6.2	561, 289	C_30_H_28_O_12_	–
13	Chalcan-flavan-3-ol dimer (isomer 4)	19.1	5,791,528	5,791,508	3.5	6.5	561	C_30_H_28_O_12_	–
14	Procyanidin trimer A-type (isomer 2)	19.4	8,631,869	8,631,829	4.6	10.3	289	C_45_H_36_O_18_	–
15	Chalcan-flavan-3-ol dimer (isomer 5)	19.8	5,791,516	5,791,508	1.3	5	561, 289	C_30_H_28_O_12_	–
16	Chalcan-flavan-3-ol dimer (isomer 6)	20.4	579,152	5,791,508	2	4	561	C_30_H_28_O_12_	–
17	(Epi)fisetinidol-(epi)catechin (isomer 1)	20.8	5,611,422	5,611,402	3.4	5.9	273	C_30_H_26_O_11_	–
18	Procyanidin A (isomer 3)	21.2	5,751,195	5,751,195	0.1	18.9	289	C_30_H_24_O_12_	–
19	(Epi)fisetinidol-(epi)catechin (isomer 2)	21.7	5,611,428	5,611,402	4.7	6.5	289, 273	C_30_H_26_O_11_	–
20	(+)-catechin	22.7	2,890,729	2,890,718	3.8	7.8	245	C_15_H_14_O_6_	[1,13,15,24,25,26,27]
21	(Epi)fisetinidol-(epi)catechin (isomer 3)	23.8	5,611,406	5,611,402	0.6	38.5	289	C_30_H_26_O_11_	–
22	(Epi)fisetinidol-(epi)catechin (isomer 4)	24.4	5,611,409	5,611,402	1.1	9.6	273	C_30_H_26_O_11_	–
23	Procyanidin A (isomer 4)	25	5,751,207	5,751,195	2	8	423	C_30_H_24_O_12_	–
24	(Epi)fisetinidol-(epi)catechin (isomer 5)	25.9	5,611,413	5,611,402	2	2.7	non fragmented	C_30_H_26_O_11_	–
25	Procyanidin A (isomer 5)	26.3	5,751,188	5,751,195	1.3	12.7	289	C_30_H_24_O_12_	–
26	Procyanidin A (isomer 6)	27.5	5,751,221	5,751,195	4.6	21.8	289	C_30_H_24_O_12_	–
27	(Epi)fisetinidol-(epi)catechin (isomer 6)	28.3	5,611,402	5,611,402	0	5	non fragmented	C_30_H_26_O_11_	–
28	(Epi)fisetinidol-(epi)catechin (isomer 7)	29.8	5,611,416	5,611,402	2.3	3	289, 273	C_30_H_26_O_11_	–
29	Procyanidin A (isomer 7)	30.7	57,512	5,751,195	0.8	14.3	289	C_30_H_24_O_12_	–
30	(Epi)fisetinidol-(epi)catechin (isomer 8)	31.1	56,114	5,611,402	0.5	11.8	245	C_30_H_26_O_11_	–
31	Procyanidin A (isomer 8)	32.7	5,751,205	5,751,195	1.7	10.7	285	C_30_H_24_O_12_	–
32	(Epi)fisetinidol-(epi)catechin (isomer 9)	33.4	5,611,418	5,611,402	2.8	7.9	289	C_30_H_26_O_11_	–
33	Quercetin rhamnosylrutinoside	34,2	7,552,041	755,204	0.2	11.9	301	C_33_H_40_O_20_	[28]
34	Rutin	36.3	6,091,476	6,091,461	0.7	14.4	301	C_27_H_30_O_16_	[28]
35	Isorhamnetin rutinoside	41.1	6,231,614	6,231,618	0.6	10.5	315	C_28_H_32_O_16_	[25]
36	Quercetin	45.8	3,010,357	3,010,354	0.9	7.4	non fragmented	C_15_H_10_O_7_	[28,29]
37	Kaempferol	49	285,041	2,850,405	1.7	11.2	non fragmented	C_15_H_10_O_6_	[30,31]

##### Monomeric Forms

The deprotonated ions (Peaks 10 and 20) at *m*/*z* 289 produced the MS^2^ fragment ions at *m*/*z* 245, which correspond to the loss of one CO_2_. These compounds were identified as (−)-epicatechin and (+)-catechin, respectively, based on the retention times and mass fragmentation comparison of [M–H]^−^ ions with authentic standards.

##### B and A-Type Oligomeric Forms

Procyanidins were identified as the main phenolic components in pine bark [1,13,26]. In agreement with data published previously, B-type procyanidins are largely procyanidins in pine bark extracts, and they contain no or less than 10% prodelphinidins [27,32]. The chemical structure of B-type oligomers was based on the presence of (epi)catechin units, which are linked by a single bond. In our study, two dimers (Peaks 6 and 7) with [M–H]^−^ ions at *m*/*z* 577 and one trimer (Peak 2) at *m*/*z* 865 have been detected. The major fragments were generated at the following *m*/*z*: *m*/*z* 289, which corresponds to deprotonated (epi)catechin; *m*/*z* 425, after the neutral loss of 152 amu (C_8_H_8_O_3_) from retro-Diels–Alder (RDA) fission of the heterocyclic C ring; and *m*/*z* 577, which corresponds to the deprotonated dimer. A-type procyanidins, which are characterized by the existence of a doubly interflavanoid linkage, have not been reported in pine bark extracts. However, in this study, A-type oligomers have been detected. In this way, Peaks 4, 5, 18, 23, 25, 26, 29 and 31 with *m*/*z* 575 and Peaks 9 and 14 with *m*/*z* 863 were tentatively identified as A-type proanthocyanidin dimers and trimers, respectively. In MS^2^, the main ions were at *m*/*z* 289, [(epi)catechin–H]^−^, and 285, [(epi)catechin–2H_2_–H]^−^, both generated by the cleavage at the interflavanoid bonds.

**Figure 3:**
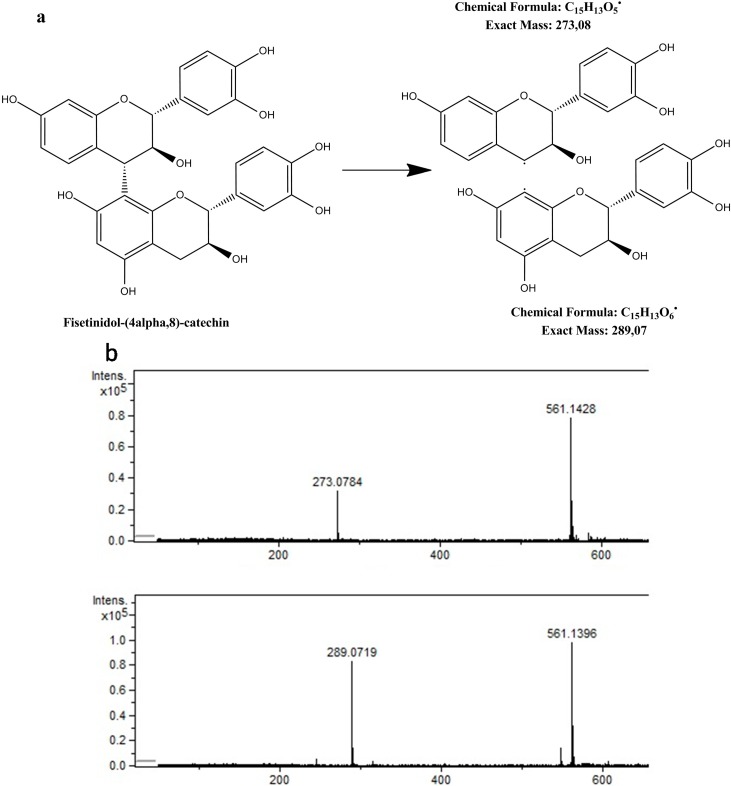
(**a**) Fragmentation pattern and (**b**) MS/MS spectra of fisetinidol-(4α,8)-catechin.

Nine isomers (Peaks 17, 19, 21, 22, 24, 27, 28, 30 and 32), with a [M−H]^−^ at *m/z* 561, were detected and generated MS^2^ fragment ions at *m*/*z* 289, 245 and 273, corresponding to deprotonated (epi)catechin, its loss of CO_2_ and deprotonated fisetinidol (Figure 3). These compounds have been identified as (epi)fisetinidol–(epi)catechin for the first time in pine bark. According to several authors, they have been detected in different kinds of bark extracts, such as *Acacia mearnsii*, *Cotinus coggygria* wood and *Mimosa* [33,34,35,36] and as gambiriin B in *Uncaria gambir* extract [37,38].

Six isomers (Peaks 8, 11, 12, 13, 15 and 16) of chalcan-flavan3-ols dimer, with a [M−H]^−^ at *m*/*z* 579, were detected in the pine bark extract. The MS^2^ spectra showed major fragment ions at 561 and 289, corresponding to the loss of H_2_O and deprotonated (epi)catechin. These compounds have also been identified in the literature as gambiriins A [37,38,39].

##### Flavonols

Peaks 33–37 were identified as flavonols and their derivatives. Peak 33, with a [M−H]^−^ at *m*/*z* 755, was tentatively identified as quercetin rhamnosylrutinoside. It showed a major fragment ion at *m*/*z* 301, which corresponded to quercetin aglycone. Peaks 34 (*m*/*z* 609) and 36 (*m*/*z* 301) were characterized as rutin and quercetin. They were confirmed by comparison with the retention times of the standards. Peak 35 had a [M−H]^−^ at *m*/*z* 623 and produced MS^2^ fragment ions at *m*/*z* 315 (isorhamnetin aglycone). This compound was identified as isorhamnetin rutinoside. Peak 37, with a [M−H]^−^ at *m*/*z* 285, was characterized as kaempferol on the basis of previously published data [30,31].

##### Other Compounds

Peak 1, which had a [M−H]^−^ at *m*/*z* 341, was tentatively identified as sucrose. Peak 3 (*m*/*z* 403) was characterized as gardenoside (iridoid).

#### 2.1.2. Green Tea Extract

A total of 35 compounds distributed in five major categories (flavan-3-ol and its derivatives, flavonols, flavanones, isoflavones and other compounds) were analyzed in the present study. Figure 2b shows the BPC of the green tea extract, and the major peaks, which were identified based on elution order, are assigned in Table 2. All of the compounds were characterized by interpreting the mass spectra obtained by QTOF-MS and also by taking into account previously reported data.

**Table 2:** Retention times and mass spectral data of the compounds characterized in green tea extract by HPLC–ESI-QTOF-MS and MS/MS in negative mode.

Peak	Proposed Compound	*RT* (min)	[M−H]^−^ Measured	[M−H]^−^ Calculated	Error (ppm)	mSigma	Fragmentation Pattern	Molecular Formula	Ref.
1	Quinic acid	5.3	1,910,562	1,910,561	0.4	7	127	C_7_H_12_O_6_	[40,41]
2	Gallic acid	12.3	1,690,143	1,690,142	0.4	4.3	125	C_7_H_6_O_5_	[17,42]
3	(Epi)gallocatechin (isomer 1)	13.7	3,050,663	3,050,667	1.1	6.6	169	C_15_H_14_O_7_	[17,40,43,44]
4	(Epi)gallocatechin-(epi)gallocatechin gallate	15.8	7,451,397	745,141	1.8	9.2	457, 169	C_37_H_30_O_17_	[40]
5	(Epi)gallocatechin gallate glucoside	16.5	6,191,308	6,191,305	0.5	4.7	457, 305	C_28_H_28_O_16_	–
6	(Epi)gallocatechin (isomer 2)	17.2	3,050,677	3,050,667	3.4	3	261, 219, 179, 165	C_15_H_14_O_7_	[17,40,43,44]
7	(−)-epicatechin	17.9	2,890,725	2,890,718	2.4	2	245	C_15_H_14_O_6_	[17,42,44]
8	Procyanidin B gallate (isomer 1)	18.9	7,291,447	7,291,461	2	15.8	577, 169	C_37_H_30_O_16_	[43,45]
9	(Epi)gallocatechin gallate (isomer 1)	20.3	4,570,789	4,570,776	2.7	2.7	169	C_22_H_18_O_11_	[17,40]
10	(Epi)gallocatechin digallate	22	6,090,911	6,090,886	4.1	5	457, 305, 169	C_29_H_22_O_15_	[40,46]
11	(+)-catechin	22.8	2,890,728	2,890,718	3.5	1.3	245	C_15_H_14_O_6_	[17,42,44]
12	(Epi)gallocatechin gallate (isomer 2)	22.9	4,570,798	4,570,776	4.8	1.4	289, 169	C_22_H_18_O_11_	[17,40]
13	(Epi)gallocatechin methyl gallate	24.1	4,710,938	4,710,933	1.2	3.3	305, 183	C_23_H_20_O_11_	[41,45,46]
14	(Epi)catechin gallate (isomer 1)	26.3	4,410,843	4,410,827	3.7	1.8	169	C_22_H_18_O_10_	[17,40]
15	(Epi)catechin gallate (isomer 2)	28.2	4,410,844	4,410,827	3.8	4.7	289, 169	C_22_H_18_O_10_	[17]
16	Procyanidin B gallate (isomer 2)	29	7,291,464	7,291,461	0.4	79.7	441, 289, 169	C_37_H_30_O_16_	[43,45]
17	Eriodictyol	29.9	2,870,565	2,870,561	1.3	9.4	non fragmented	C_15_H_12_O_6_	[17]
18	(Epi)catechin methyl gallate	30.5	4,550,987	4,550,984	0.6	15.8	289, 183	C_23_H_20_O_10_	[47]
19	Epiafzelechin gallate	31.3	425,088	4,250,878	1.1	8.4	169	C_22_H_18_O_9_	[48]
20	Myricetin glucoside	31.8	4,790,815	4,790,831	3.2	5.8	317	C_21_H_20_O_13_	[40,44]
21	Genistein glucoside (isomer 1)	32.2	4,310,985	4,310,984	0.3	4.6	269	C_21_H_20_O_10_	–
22	Genistein glucoside (isomer 2)	34.3	4,310,981	4,310,984	0.6	9.9	269	C_21_H_20_O_10_	–
23	Rutin	36.4	6,091,486	6,091,461	4.1	7.3	463	C_27_H_30_O_16_	[40,45]
24	Naringenin	37.8	271,062	2,710,612	3.1	2.7	non fragmented	C_15_H_12_O_5_	[17,49]
25	Kaempferol glucosylrutinoside	38.6	7,552,056	755,204	2.1	12.5	447, 285	C_33_H_40_O_20_	[44,45]
26	Kaempferol-glucoside	39.5	4,470,937	4,470,993	1	9.8	285	C_21_H_20_O_11_	[44,45]
27	Myricetin	39.9	3,170,308	3,170,303	1.6	12.3	non fragmented	C_15_H_10_O_8_	[40,49]
28	Kaempferol rutinoside	40.5	593,151	5,931,512	0.3	3.6	447	C_27_H_30_O_15_	[45]
29	Morin	43.4	3,010,355	3,010,354	0.4	4.7	non fragmented	C_15_H_10_O_7_	[50]
30	Theaflavin gallate	44.5	7,151,309	7,151,305	0.6	16.3	563, 545	C_36_H_28_O_16_	[17,40,43]
31	Theaflavin digallate	44.9	8,671,387	8,671,414	3.1	25.1	715, 563, 545	C_43_H_32_O_20_	[17,40,43]
32	Theaflavin	45.3	5,631,187	5,631,195	0.5	7.3	545	C_29_H_24_O_12_	[17,40,43]
33	Quercetin	45.8	3,010,362	3,010,354	2.6	2.9	non fragmented	C_15_H_10_O_7_	[45,49]
34	Kaempferol-coumaryl-glucoside	46.9	5,931,293	5,931,301	1.3	17.4	447	C_29_H_24_O_12_	[45]
35	Kaempferol	49	2,850,418	2,850,405	4.7	1.5	non fragmented	C_15_H_10_O_6_	[45,49]

##### Flavan-3-ol and Its Derivatives

The main flavan-3-ols found were gallate ester derivatives. The deprotonated ions at *m*/*z* 305 (Peaks 3 and 6) generated the MS^2^ fragment ions at *m*/*z* 261, 219, 179, 169 and 165, which are in keeping with the loss of one CO_2_, C_4_H_6_O_2_, C_6_H_6_O_3_, gallic acid and C_7_H_8_O_3_, respectively. The loss of C_4_H_6_O_2_ was due to the cleavage of the A ring of flavan-3-ol. The loss of C_6_H_6_O_3_ resulted from heterocyclic ring fission (HRF). The loss of C_7_H_8_O_3_ occurred through retro-Diels–Alder (RDA) fission. Theses peaks were identified as (epi)gallocatechin isomers [43]. Peak 5 had a [M−H]^−^ at *m*/*z* 619. Its MS^2^ spectrum showed fragment ions at *m*/*z* 457 (corresponding to (epi)gallocatechin gallate)) and 305 (corresponding to (epi)gallocatechin)). It was identified as (epi)gallocatechin gallate glucoside. Peaks 9 and 12, with [M−H]^−^ at *m*/*z* 457 ((epi)gallocatechin gallate), produced the MS^2^ fragment ions at *m*/*z* 289 and 169, which corresponded to the deprotonated ion of (epi)catechin and gallic acid, respectively. Peak 10 was identified as (epi)gallocatechin digallate based on the mass spectra data. This compound produced the MS^2^ fragment ions at *m*/*z* 457, 305 and 169, which corresponded to (epi)gallocatechin gallate, (epi)gallocatechin and gallic acid, respectively. Peak 13 had the [M−H]^−^ at *m*/*z* 471, with product ions at *m*/*z* 305 and 183, corresponding to the cleavage of (epi)gallocatechin and methylgalloyl moiety. It was identified as (epi)gallocatechin methyl gallate. The deprotonated ions at *m*/*z* 441 (Peaks 14 and 15) produced the MS^2^ fragment ions at *m*/*z* 289 and 169, corresponding to the deprotonated ions of catechin (or epicatechin) and gallic acid, respectively. They were identified as (epi)catechin gallate isomers. Peak 18, which was characterized as (epi)catechin methyl gallate, had a [M−H]^−^ at *m*/*z* 455. The product ions were obtained at *m*/*z* 289 and 183, consistent with the cleavage of (epi)catechin and a methylgalloyl moiety [47]. Peak 19 was identified as epiafzelechin gallate according to previous reports and the interpretation of the mass spectra obtained [48]. Its MS^2^ spectrum showed a major fragment ion at *m*/*z* 169 (gallic acid).

The deprotonated ion at *m*/*z* 289 produced the MS^2^ fragment ions at *m*/*z* 245, corresponding to the loss of one CO_2_. Peaks 7 and 11 were identified as (−)-epicatechin and (+)-catechin based on the retention times and mass fragmentation comparison of [M−H]^−^ ions with authentic standards. Peak 4, with a [M−H]^−^ at *m*/*z* 745, showed MS^2^ fragment ions at *m*/*z* 457 ((epi)gallocatechin gallate)) and 169 (gallic acid). This compound was tentatively identified as (epi)gallocatechin-(epi)gallocatechin gallate. Peaks 8 and 16 were detected at *m*/*z* 729 and were tentatively assigned as galloyl(epi)catechin-(epi)catechin isomers. This compound produced the MS^2^ at *m*/*z* 577 (loss of a galloyl residue), at *m*/*z* 441 ((epi)catechin gallate)), at *m*/*z* 289 (deprotonated (epi)catechin)) and at *m*/*z* 169 (deprotonated gallic acid).

Peaks 30, 31 and 32 were identified as theaflavin and its derivatives (gallate and digallate). These compounds showed the [M−H]^−^ at *m*/*z* 715, 867 and 563, which corresponded to theaflavin gallate, theaflavin digallate and theaflavin, respectively. The product ions were obtained at *m*/*z* 715 (theaflavin gallate), 563 (theaflavin aglycone) and 545 (loss of a 18 amu, which was consistent with the cleavage of one H_2_O) [43].

##### Flavonols

Peaks 20, 23, 25–29 and 33–35 were identified as flavonols and derivatives. Peaks 20 and 27, with [M−H]^−^ at *m*/*z* 479 and 317, respectively, were tentatively identified as myricetin glucoside and myricetin, respectively [40]. Peaks 23 (*m*/*z* 609) and 33 (*m*/*z* 301) were characterized as rutin and quercetin, respectively. They were confirmed by comparison with the retention times of the standards. Considering the elution order, Peaks 25, 26, 28, 34 and 35, with [M−H]^−^ at *m*/*z* 755, 447, 593, 593 and 285, respectively, were identified as kaempferol and its derivatives. Product ion spectra of these peaks showed fragment ions at *m*/*z* 447 and 285, corresponding to kaempferol glucoside and kaempferol aglycone, respectively. On the basis of the mass spectra and previously published data, these compounds were tentatively identified as kaempferol glucosylrutinoside, kaempferol glucoside, kaempferol rutinoside, kaempferol coumarylglucoside and kaempferol [45]. Peak 29 had a [M−H]^−^ at *m*/*z* 301 and was tentatively identified as morin [50].

##### Flavanones

Two flavanones, with [M−H]^−^ at *m*/*z* 287 (peak 17) and 271 (peak 24), were characterized as eriodictyol and naringenin, respectively [17].

##### Isoflavones

Genistein glucoside isomers (peaks 21 and 22) were found at *m*/*z* 431. The product ion spectra of these peaks showed a major fragment ion at *m*/*z* 269, corresponding to genistein aglycone.

##### Other Compounds

Peak 1 had a [M–H]^−^ at *m*/*z* 191 and was identified as quinic acid, for which the most important fragment appeared at *m*/*z* 127 ([M–H–CO–H_2_O]^−^). Peak 2, with a [M−H]^−^ at *m*/*z* 169 and MS^2^ fragment ion at *m*/*z* 125 (decarboxylation of galloyl group), was identified as gallic acid according to the literature [17] and confirmed by comparison with the retention time of the standard.

### 2.2. Total Phenolic and Flavan-3-ol Contents and in Vitro Antioxidant Activities of Pine Bark and Green Tea Extracts

The antioxidant activity of polyphenols depends on the arrangement of the functional groups around the nuclear structure. Pine bark and green tea extracts are mainly composed of “bricks” of the flavan-3-ols, catechin and epicatechin, linked together into different lengths [13]. These compounds were found to be efficient scavengers of free radicals in a number of *in vitro* systems. The principal naturally occurring catechins in green tea leaves are with galloyl groups [51]. The presence of an *ortho*-dihydroxyl group in the B-ring has been shown to be important for the radical scavenging abilities of tea catechins. The addition of a gallate moiety at the 3 position of the C-ring increases the radical scavenging effectiveness of catechins in a number of systems [52].

Since the methods used to measure total phenolic and flavan-3-ol contents and antioxidant activities are extremely dependent on the reaction conditions and the substrates or products, not all methods yield the same values for activity [53]. Prior to the measurement of the antioxidant activity, the total phenolic and flavan-3-ol contents of pine bark and green tea extracts were quantified using the Folin–Ciocalteu method and vanillin assays, respectively. The obtained values for each assay are shown in Table 3. On the basis of the dry weight, the total phenolic contents were 847.62 ± 39.74 mg of gallic acid equivalents (GAE) g-1 (pine bark) and 835.23 ± 50.31 mg GAE g-1 (green tea), and total flavan-3-ol contents were 883.33 ± 76.38 mg of (+)-catechin equivalents (CE) g-1 (pine bark) and 906.25 ± 150.26 mg CE g-1 (green tea). According to Ku *et al.*, total polyphenol content in various pine bark varieties ranged from 111 to 862 mg/g [54], and to according Gramza *et al.*, total polyphenol content in tea extracts varied between 245.8–837.6 mg/g [55].

**Table 3:** Values for different antioxidant measurements performed with pine bark and green tea extracts. Values are expressed as the mean ± SD.

Assays	Pine Bark	Green Tea
Folin–Ciocalteu ^a^	847.62 ± 39.74 [54,56]	835.23 ± 50.31 [55]
Vanillin assay ^b^	883.33 ± 76.38	906.25 ± 150.26
TEAC ^c^	5.72 ± 0.78	9.66 ± 1.27 [57]
FRAP ^d^	4.83 ± 0.15	8.4 ± 0.4
ORAC ^c^	8.4 ± 0.4 [56]	7.58 ± 0.57 [57]

^a^ Expressed in mg gallic acid equivalents g^−1^ extract (dw); ^b^ expressed in mg (+)-catechin equivalents g^−1^ extract (dw); ^c^ expressed in mmol Trolox equivalents g^−1^ extract (dw); ^d^ expressed in mmol FeSO_4_ equivalents g^−1^ extract (dw).

Different *in vitro* methods were performed to determine the antioxidant activity of pine bark and green tea extracts. TEAC and FRAP are based on a single-electron transfer mechanism (ET). TEAC has been applied to establish the antioxidant properties of components in a large variety of food samples [58], and FRAP is specially indicated for determining the antioxidant capacity of biological samples [59]. The ORAC assay is performed in order to test the capacity of the extracts to quench peroxyl radicals. ORAC determination is based on a hydrogen atom transfer assay and has become one of the most widely accepted methods for measuring the antioxidant capacity of food, botanical and biological samples [58].

Table 3 lists the antioxidant capacities by TEAC and FRAP of both concentrated extracts. According to the single-electron transfer-based methods, TEAC and FRAP assays, the values for the whole pine bark extract were 5.72 ± 0.78 mmol of Trolox equivalents (TE) g^−1^ and 4.83 ± 0.15 mmol FeSO_4_ equivalents (FE) g^−1^, respectively. For the whole green tea extract, the values were 9.66 ± 1.27 mmol TE g^−1^ and 8.4 ± 0.4 mmol of FeSO_4_ equivalents (FE) g^−1^, respectively. By the ORAC assay, the values were 8.4 ± 0.4 mmol TE g^−1^ for the pine bark extract and 7.58 ± 0.57 mmol TE g^−1^ for the green tea extract. Seeram *et al.* have determinated the antioxidant activities of green tea dietary supplements by TEAC and ORAC. These values ranged from 1.87 to 15.340 and from 1.66 to 13.690 mmol TE g^−1^, respectively [57]. The antioxidant activity of different bark extracts was analyzed by Legault *et al.* The ORAC values ranged from 2.4 to 29 mmol TE/g [56].

By comparing all of our assays, both extracts showed high values of antioxidant activities and total phenolic and flavan-3-ol contents. This could be a result of our samples being rich in flavan-3-ol, mainly the oligomeric forms. Other sources, which have been reported to contain oligomeric flavan-3-ols (*i.e*., cocoa), showed similar antioxidant capacity values [7]. These results showed that, for these two extracts, the green tea extract was a better antioxidant by electron transfer-based mechanisms, and pine bark extract was better by hydrogen atom transfer-based mechanisms. However, as shown in Table 3, total phenolic and total flavan-3-ol contents were similar and could not explain the differences in the antioxidant capacity, demonstrating that these two values only can be used as indicators. To understand why green tea and pine bark extracts were powerful antioxidants by different mechanisms, an in-depth characterization is needed to identify the phenolic composition of each. According to Table 1 and Table 2, the green tea extract was rich in gallic acid and gallate derivatives. In addition to the antitumor [1] and antimicrobial activities [2], gallic acid, as well as gallate derivatives have been described to have notable antioxidant activity by ET-based mechanisms [3,4,5,6,7]. This is, in part, due to the three hydroxyl groups in its phenolic ring [8]. On the other hand, pine bark extract was rich in procyanidins. These compounds have anti-inflammatory [9,10] and anticancer activities [11,12], as well as antioxidant properties, which are commonly determined by HAT-based mechanisms [13,14,15,16]. These findings demonstrated that polyphenols (even if they are considered to be universal antioxidants) act under different mechanisms based on their structure [17,18,19,20]. A comparison of these results with previous reports does not yield useful or tenable information due to differences in the nature of the samples and pre-concentration technologies, extraction systems and assay methodologies.

## 3. Experimental Section

### 3.1. Chemicals

All chemicals were of analytical reagent grade and used as received. Acetic acid and acetonitrile for UHPLC were purchased from Fluka, Sigma–Aldrich (Steinheim, Germany) and Lab-Scan (Gliwice, Sowinskiego, Poland), respectively. Solvents were filtered using a Solvent Filtration Apparatus 58061 (Supelco, Bellefonte, PA, USA). Dimethyl sulfoxide (DMSO) was purchased from Panreac (Barcelona, Spain).Water was purified by a Milli-Q system from Millipore (Bedford, MA, USA).

The standards, procyanidin A2, (+)-catechin, (−)-epicatechin, gallic acid, quercetin and rutin, were purchase either from Fluka, Sigma-Aldrich (Steinheim, Germany) or Extrasynthese (Genay Cedex, France).

The reagents used to measure the antioxidant capacity and total phenolic/flavanol-3-ol content, AAPH (2,2'-azobis-2-methyl-propanimidamide, dihydrochloride), TPTZ (1,3,5-triphenyltetrazolium chloride), ABTS (2,2'-azinobis (3-ethylbenzothiazoline-6-sulphonate)), Trolox (6-hydroxy-2,5,7,8-tetramethylchroman-2-carboxylic acid), fluorescein, potassium persulfate, ferric sulfate, Folin-Ciocalteu reagent, (+)-catechin and vanillin, were purchased from Sigma–Aldrich (St. Louis, MO, USA). Dehydrated sodium phosphate, trihydrated sodium acetate, sodium acetate, ferric chloride, hydrochloric acid, sodium carbonate and gallic acid were obtained from Panreac (Barcelona, Spain).

### 3.2. Sample Preparation

Concentrated pine bark and green tea extracts (Nutrafur, Spain) were used in this study. The polyphenols from whole extracts were analytically characterized using a 10 mg/mL solution of pine bark or green tea extracts. Briefly, 10 mg of these extracts were dissolved in 1 mL of DMSO. The sample was sonicated for 5 min, vortexed for 1 min, centrifuged for 5 min at 7700× *g* and then filtered through a 0.25 mm filter before the HPLC analysis.

### 3.3. Instrumentation

Analytical characterizations of pine bark and green tea extracts were performed using an Agilent 1200 series rapid-resolution LC system (Agilent Technologies, Palo Alto, CA, USA) equipped with a binary pump, an autosampler and a diode array detector (DAD). The HPLC system was coupled to a quadrupole time-of-flight mass spectrometer (QTOF) mass spectrometer (Bruker Daltonics, Bremen, Germany) equipped with an electrospray ESI interface (model G1607A from Agilent Technologies, Palo Alto, CA, USA). Fluorescence (ORAC) and absorbance (Folin-Ciocalteu assay, vanillin assay, FRAP and TEAC) measurements were carried out on a Synergy Mx Monochromator-Based Multi-Mode Micro plate reader (Bio-Tek Instruments Inc., Winooski, VT, USA) using 96-well polystyrene microplates.

### 3.4. Chromatographic, UV and Spectrophotometric Conditions

The compounds from pine bark and green tea extracts were separated at room temperature using a Zorbax Eclipse Plus C18 column (1.8 μm, 150 mm × 4.6 mm). The mobile phases consisted of 0.5% acetic acid (A) and methanol (B). The following multi-step linear gradient was applied: 0 min, 0% B; 5 min, 25% B; 15 min, 35% B; 20 min, 39% B; 38 min, 60% B; 40 min, 70% B; 42 min, 80% B; 44 min, 100% B; 46 min, 0% B; and 48 min, 0% B. The initial conditions were held for 10 min. The injection volume was 10 μL, and the flow rate was 0.3 mL/min. For the spectrophotometric conditions for antioxidant assays, the excitation and emission wavelengths were 485 and 520 nm, respectively, for the ORAC assay. The absorbance wavelengths for Folin-Ciocalteu, vanillin, FRAP and TEAC assays were 760, 500, 593 and 734 nm, respectively.

### 3.5. ESI-QTOF-MS Detection

The HPLC system was coupled to a QTOF mass spectrometer equipped with an ESI interface operating in negative ion mode using a capillary voltage of +3.5 kV. The other optimum values of the source parameters were: drying gas temperature, 220 °C; drying gas flow, 9 L/min; and nebulizing gas pressure, 2.5 bar. The detection was performed for a mass range of 50–1200 *m*/*z*.

The accurate mass data of the molecular ions were processed through the Data Analysis 4.0 software (Bruker Daltonics, Bremen, Germany), which provided a list of possible elemental formulas using Generate Molecular Formula Editor. This uses a CHNO algorithm, which provides standard functionalities, such as minimum/maximum elemental range, electron configuration and ring-plus double-bond equivalents, as well as a sophisticated comparison of the theoretical with the measured isotope patterns (σ value) for increased confidence in the suggested molecular formula [60]. The widely accepted accuracy threshold for the confirmation of elemental compositions was established at 5 ppm [61]. Even with a very high mass accuracy (<3 ppm in most of the cases), many chemically possible formulae were determined depending on the mass regions considered.

Therefore, high mass accuracy alone is not sufficient to exclude enough candidates with complex elemental compositions. The use of isotopic abundance patterns as a single further constraint removes >95% of the false candidates. This orthogonal filter can reduce several thousand candidates to only a small number of molecular formulas.

During the development of the HPLC method, external instrument calibration was performed using a 74900-00-05 Cole Palmer syringe pump (Vernon Hills, IL, USA) directly connected to the interface, with a sodium acetate cluster solution passing through, containing 5 mM sodium hydroxide and 0.2% acetic acid in water:isopropanol (1:1, *v*/*v*). The calibration solution was injected at the beginning of each run, and all of the spectra were calibrated prior to compound identification.

### 3.6. Total Phenolic and Flavan-3-ol Contents

The total phenolic content was measured by the Folin–Ciocalteu method reported by [62], with some modifications. The extracts were dissolved in methanol (different concentrations of extracts were tested). Then, 10 μL aliquots were mixed with 50 μL of Folin-Ciocalteu reagent, 150 μL of 20% (*w*/*v*) sodium carbonate solution and 600 μL water. After 2 h of incubation at room temperature in the dark, 200 μL of the mixture was transferred into a well of the microplate, and the absorbance was read at 760 nm against a blank in a microplate spectrophotometer reader (BioTek, Winooski, VT, USA). The phenol content was calculated based on the calibration curves of gallic acid and expressed as mg GAE/g of dry matter. Measurements were made in triplicate.

Both extracts were analyzed for total flavan-3-ol content using a method described in [63], with some modifications. For the analysis, a working solution of 1% vanillin in methanol and 10% HCl in methanol (1:1, *v*/*v*) was prepared daily. The extract was dissolved in methanol (different concentrations were tested). Then, 100 μL aliquots were mixed with 1 mL of the previously prepared vanillin reagent. The mixture was allowed to react for 30 min at a room temperature. After that, 200 μL of the mixture were transferred into a well of the microplate, and the absorbance was read at 50 nm against a blank in a microplate spectrophotometer reader (BioTek). The blank was prepared by replacing the 100 μL samples or standard with methanol. Flavan-3-ol content was calculated based on the calibration curves of (+)-catechin and expressed as mg CE/g of dry matter. Measurements were made in triplicate.

### 3.7. Antioxidant Capacity Assays

The TEAC assay, which measures the reduction of the radical cation of 2,2'-azinobis-(3-ethylbenzothiazoline-6-sulphonate) (ABTS) by antioxidants, was performed by using a previously described method [6,7,64,65]. TEAC values were calculated using Trolox as the standard. The FRAP assay was carried out following the method described by Benzie and Strain, Cádiz-Gurrea *et al.* and Morales-Soto *et al.* [6,7,59,65]. FRAP values were calculated using FeSO_4_·7H_2_O as the standard. To assay the capacity of the extracts to scavenge peroxyl radicals, a validated ORAC method was used [66] with the modifications developed by Laporta *et al.*, Cádiz-Gurrea *et al.* and Morales-Soto *et al.* [6,7,64,65]. The final ORAC values were calculated using a regression equation between the Trolox concentration and the net area of the fluorescence decay curve (area under curve, AUC), as previously described in Laporta *et al.* [64]. Measurements were made in triplicate.

## 4. Conclusions

In the present study, HPLC–ESI-QTOF-MS has been confirmed as a powerful analytical technique for separating and detecting phenolic and other polar compounds in concentrated pine bark and green tea extracts. With this method, 37 compounds were tentatively identified in pine bark extract and 35 compounds in green tea extract based on their chromatographic retention, MS data and MS/MS fragmentation pattern. The most representative groups of compounds tentatively identified were flavan-3-ols (oligomeric forms). Of these compounds, (epi)fisetinidol-(epi)catechin isomers and other chalcan-flavan-3ol isomers have been tentatively identified for the first time in pine bark.

These extracts possess significant antioxidant capacity to reduce peroxyl radicals determinated by the ORAC assay. Moreover, both extracts show a strong capacity to donate electrons by FRAP and TEAC assays. Additionally, they both had high phenolic and flavan-3-ol contents.
